# One-step laparoscopic cholecystectomy with common bile duct exploration and stone extraction versus two-step endoscopic retrograde cholangiography with stone extraction plus laparoscopic cholecystectomy for patients with common bile duct stones: a randomised feasibility and pilot clinical trial—the preGallStep trial

**DOI:** 10.1186/s40814-023-01251-z

**Published:** 2023-02-06

**Authors:** Anders Kirkegaard-Klitbo, Daniel Mønsted Shabanzadeh, Markus Harboe Olsen, Jane Lindschou, Christian Gluud, Lars Tue Sørensen

**Affiliations:** 1 Digestive Disease Centre K, Copenhagen University Hospital, Bispebjerg, Copenhagen, Denmark; 2 Digestive Disease Centre, Copenhagen University Hospital, Hvidovre, Copenhagen, Denmark; 3 Copenhagen Trial Unit, Centre for Clinical Intervention Research, The Capital Region, Copenhagen University Hospital, Rigshospitalet, Copenhagen, Denmark; 4 Department of Neuroanaesthesiology, The Neuroscience Centre, Copenhagen University Hospital, Rigshospitalet, Copenhagen, Denmark; 5 Department of Regional Health Research, The Faculty of Health Sciences, University of Southern Denmark, Odense, Denmark; 6 Department of Clinical Medicine, The Faculty of Health and Medical Sciences, Copenhagen University, Copenhagen, Denmark

**Keywords:** Common bile duct stones (CBDS), Laparoscopic common bile duct exploration (LCBDE), Endoscopic retrograde cholangiography (ERC), Complications, Feasibility trial, Pilot trial, Randomised trial, Clavien-Dindo classification

## Abstract

**Background:**

Endoscopic retrograde cholangiography (ERC) with stone extraction and papillotomy with subsequent laparoscopic cholecystectomy—the two-step approach—is the standard treatment of common bile duct stones in many countries. However, ERC is associated with a high risk of complications and more than half of patients require multiple ERCs. Meta-analyses of randomised clinical trials find no major differences of the two-step approach in comparison with laparoscopic cholecystectomy with intraoperative laparoscopic stone clearance—the one-step approach. Currently, there are insufficient data to ascertain superiority.

**Methods:**

The preGallstep trial is an investigator-initiated, multicentre randomised feasibility and pilot clinical trial with blinded outcome assessment. Eligible patients are patients with common bile duct stones (identified by magnetic resonance cholagiopancreatography), age 18 years or above with the possibility to perform both interventions within a reasonable time. We intent to randomise 150 participants allocated 1:1. The experimental intervention is the one-step approach. This consists of laparoscopic common bile duct exploration plus laparoscopic cholecystectomy. The control intervention is the two-step approach which consists of ERC plus sphincterotomy (first step) and subsequent laparoscopic cholecystectomy (second step). Feasibility outcomes include the proportion of eligible patients not wanting to participate, reasons for rejection to participate, difficulties during the informed consent procedure, difficulties with randomisation, difficulties with data management, difficulties with blinding patient charts and forms and difficulties with maintaining blinding for the outcome assessors. The primary pilot outcome is the proportion of participants with at least one postoperative complication according to the Clavien-Dindo score grade II and above until 90 days after randomisation. This outcome will be used for a future sample size calculation of a larger pragmatic trial. Further, a range of clinical explorative outcomes will be assessed.

**Discussion:**

As no sample size is estimated in this trial, there is a risk of wrongly assessing the effect on the patient-related outcome. The surgical procedures cannot be blinded. However, blinding will be employed in all other aspects of the trial, including the establishment of a blinded outcome adjudication committee with three independent assessors. Heterogeneity in screening, randomisation, diagnostics, treatment procedures, interventions and follow-up across trial sites may cause challenges in conducting a larger pragmatic trial. To monitor inter-site differences, we have implemented a central data monitoring scheme.

**Trial registration:**

ClinicalTrials.gov identification: NCT04801238, Registered on 16 March 2021

## Background

In Denmark alone, more than 7500 cholecystectomies are conducted each year making it one of the most common general surgical procedures [1]. Common bile duct gallstones (CBDS) are found in up to 18% of patients undergoing cholecystectomy [2]. The *two-step approach* including endoscopic retrograde cholangiography (ERC) with stone extraction and papillotomy plus a subsequent laparoscopic cholecystectomy has become the standard treatment of CBDS [3]. However, ERC is associated with a high risk of postoperative pancreatitis and more than half of patients may require multiple ERCs due to retained stones [2, 4].

Recent randomised clinical trials have shown comparable proportions of successful CBDS clearance, risk of short-term postoperative complications (e.g. perioperative bleeding, postoperative infections and damage to the biliary structures) and mortality between the two-step versus the *one-step approach* [5–10]*.* The one-step approach entails laparoscopic cholecystectomy with intraoperative common bile duct exploration and stone clearance. A one-step approach may seem beneficial compared with the two procedures separated by a couple of days or weeks. However, the one-step procedure requires special equipment, special surgical training and often a longer duration of the operation. Furthermore, anatomical variations in the biliary system, the number of stones, or the size of stones may influence on the possibility of retrieving the stones. Recently published meta-analyses and a systematic review with meta-analyses find that the one-step approach may be superior to the two-step approach in terms of safety, including perioperative complications, conversion rate to other procedures, CBDS clearance, hospital stay, operative time, in-hospital costs and stone recurrence [11–13]. However, these meta-analyses selected only English language studies; included only fully published articles; included trials with questionable randomisation, lack of confirmation of CBDS, lack of blinding and lack of follow-up; and did not assess patient-reported outcome measures (PROMs) [11–13]. Only 1/14 randomised clinical trials [5–10, 14–21] assessed systematically postoperative complications by the Clavien-Dindo classification [21]. The Clavien-Dindo score grades the postoperative complications according to the requirement of treatment needed [22]. The five-grade scale contains grade I defined by a mild deviation during the postoperative cause; grade II as requiring pharmacological drugs or blood transfusion; grade III as requiring surgical, endoscopic or radiological intervention; grade IV as organ failure requiring intensive care unit (ICU) treatment; and finally grade V as death. Trial Sequential Analysis (TSA) [23–26] was only carried out in one of the meta-analyses [12]. This TSA focused on the proportion of successful CBDS clearance and did not include trials with only clinical suspicion of CBDS without radiology confirmation. The overall conclusion of the systematic review suggests a potential for a true superiority of the one-step approach, but the TSA is still underpowered.

**Fig. 1:**
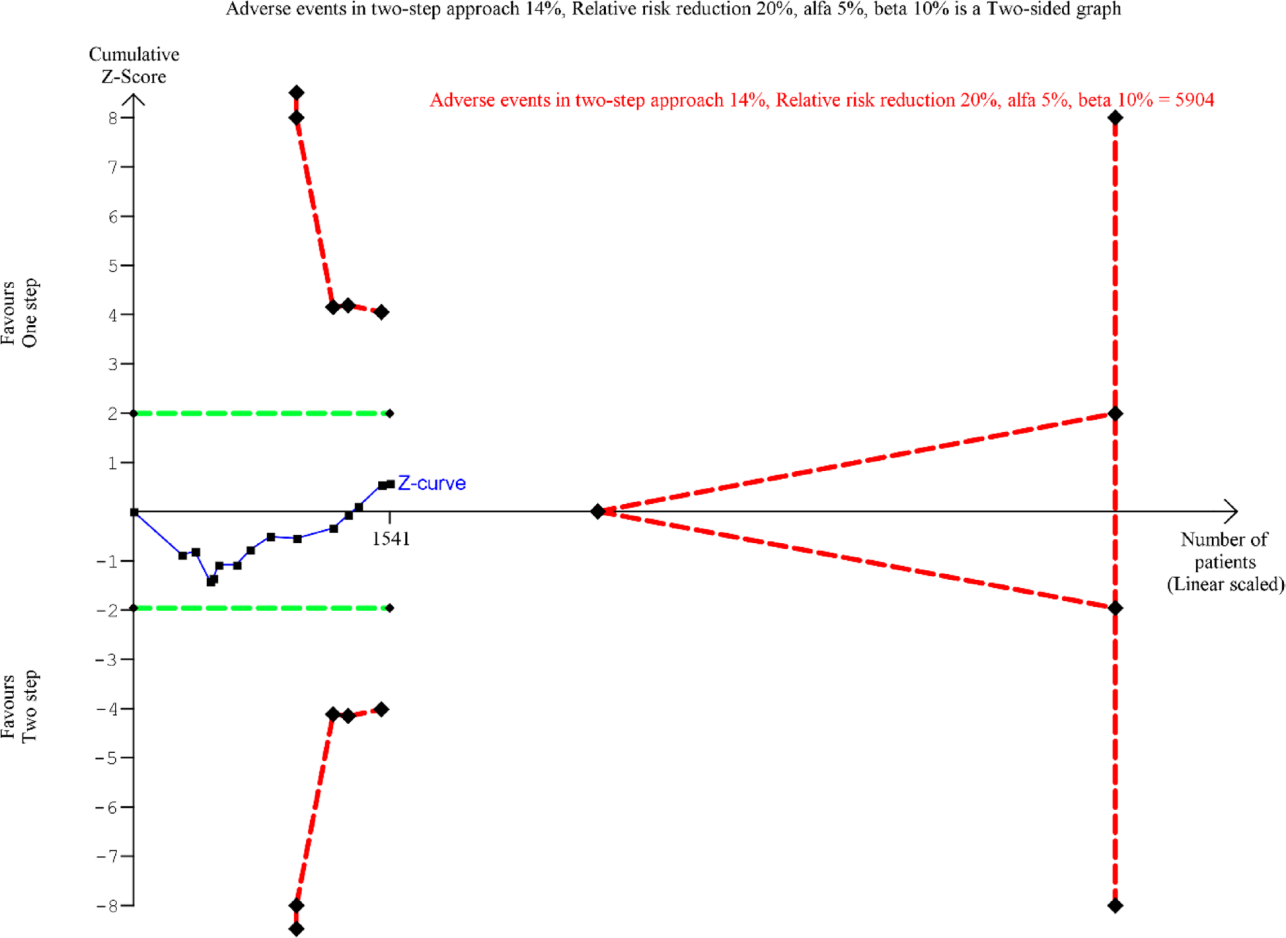
Trial sequential analysis. 1541 patients are included in 14 RCTs. TSA is conducted with an α of 5% and a β of 10%. The risk of complications is set to 14% and the relative reduction of risk to 20%. The necessary sample size to reject or accept such an intervention effect is 5904 patients

Two authors (AKK and DMS) carried out a systematic literature search of The Cochrane Library, MEDLINE and Embase in February 2022 and found three new randomised clinical trials since the publication of the meta-analyses by Singh and Kilambi in 2018 [12]. All 14 randomised clinical trials were included in a new TSA [5–10, 14–21] (Fig. 1). Our primary outcome was to assess the proportions of participants in each intervention group with adverse events according to the Clavien-Dindo classification. Based on the TSA, with a relative risk reduction of 20%, *α* at 0.05, *β* at 0.10 and diversity of 0%, the total meta-analytic sample size required was 5904 participants for demonstrating a difference between the two surgical approaches on postoperative complications. Previous trials only included 1541 patients in total. Our TSA demonstrates that more patients are needed in randomised trials to accept futility or declare one of the approaches as superior.

To date, no large-scale randomised clinical trial has been conducted, presumably because of the complexity of the trial setup. More importantly, heterogenicity of previous trials is present in the form of exclusion of randomised patients without CBDS at intraoperative cholangiography [8, 17, 19, 20], exclusion of patients that did not complete the protocolled treatments [7, 8, 15], randomisation of patients with only a clinical suspicion of CBDS [8, 14, 15, 17, 19–21] and only a few trials randomised patients with CBDS proven by magnetic resonance cholangio-pancreaticography (MRCP) or endoscopic ultrasound (EUL) [5–8, 18]. Before conducting such a large-scale pragmatic trial, it is crucial to investigate the feasibility and the practical approaches needed. Therefore, we wish to conduct a feasibility and pilot trial—*the preGallstep trial*—prior to conducting a large pragmatic randomised clinical trial.

Feasibility outcomes include the following:

- Consumption of manpower
- Difficulties getting the first participant randomised at each clinical site
- Reasons for rejection to participate
- Reasons for not being eligible for inclusion
- Difficulties during the informed consent procedure
- Difficulties with randomisation
- Difficulties in data management
- Difficulties with blinding patient charts and forms;
- Difficulties in maintaining blinding for the outcome assessors
- Proportion of eligible patients not wanting to participate

Analysis of these outcomes will help us in planning a larger confirmatory trial comparing one- and two-step procedures in the treatment of common bile duct stones.

## Methods/design

### Objectives

The main objective of this randomised feasibility and pilot clinical trial is to assess the feasibility of conducting this trial and secondary to estimate the proportions of participants in each interventional group with postoperative complications according to the Clavien-Dindo classification grade II or more. The purpose is to obtain estimates for a sample size estimation of a pragmatic randomised clinical trial. This feasibility and pilot trial will, furthermore, explore the effects of the interventions on CBDS clearance failure, number of procedures needed, length of hospital stay, procedure-specific outcomes, quality of life and liver biochemistry.

### Trial design

The preGallstep trial is conducted according to the 2013 SPIRIT statement [27]. The preGallStep trial is an investigator-initiated, multicentre randomised parallel group, feasibility and pilot clinical trial, with blinded outcome assessment comparing the one-step versus the two-step approach. Patients not included in the trial or withdrawing their consent will be offered the course of treatment deemed most appropriate by the attending surgeon. The first participant was included and randomised on 22 April 2021.

### Participant timeline

Eligible patients can enter the trial through a variety of ways. While the most frequent in-hospital entry is through the emergency department, a few patients with CBDS will also be referred to the out-patient clinic from other departments or from private practices.

Patients will be offered enrolment into the trial if all inclusion and no exclusion criteria are met (see the “Criteria for eligibility”). Patients will be informed of the trial by the attending surgeon and offered participation. Written and oral informed consent shall be obtained, and baseline data collected. Randomisation will be obtained by the trial site investigator. Short form 36 (SF-36), a quality-of-life questionnaire, will be completed prior to the surgical interventions. The designated intervention will be carried out within 24 h of randomisation or possibly twice a week if local facilities are unable to provide operating facilities within 24 h of randomisation. If no immediate complications occur during or after the interventions, the patients will be discharged within 24 h. Blood samples including serum amylase will be drawn 24 to 36 h postoperatively. A 90-day follow-up will be performed to assess patient-related outcomes. Radiographic imaging will be performed only if clinically indicated. Registration of lost to follow-up and reasons will also be assessed.

### Criteria for eligibility

#### Inclusion criteria

- CBDS identified by MRCP
- Age 18 years or older
- Ability to perform both interventions within a reasonable time
- Informed consent

#### Exclusion criteria

- Common bile duct cysts
- Pancreatic/biliary/hepatic malignancies
- Prior cholecystectomy or sphincterotomy
- Chronic pancreatitis
- Cholangitis grade 3 according to the Tokyo Guidelines [28] (cholangitis with organ dysfunction)
- Previous gastric-bypass surgery or other previous surgery preventing ERC, LC, or LCBDE
- Pregnancy
- If the patient is unable to give an informed consent

### Randomisation

Participants will be randomised at the allocation ratio 1:1. Randomisation will be performed centrally at the Copenhagen Trial Unit (Copenhagen, Denmark) using a computer-generated allocation sequence with a varying block size concealed from the investigators. The allocation sequence will be stratified by the trial site. Copenhagen Trial Unit will generate the allocation sequence, and participants are enrolled using a web-based system developed by the unit.

### Blinding

The obvious advantages of the one-step approach compared with the two-step approach are the fewer procedures required for CBDS clearance and removal of the gallbladder. Due to the nature of the surgical and endoscopic interventions, blinding of patients or surgeon/endoscopist is not possible in this trial. To prevent dropout due to patient preferences, patients are blinded from assigned intervention until 72 h prior to surgery. Most of the outcomes are dependent on the physician’s clinical assessment. However, we will engage a blinded adjudication committee of three independent experts who will examine medical charts from randomisation to 90 days after the first surgical intervention for outcome assessment. The medical charts presented to the adjudication committee will be blinded for any phrases related to the intervention, and the committee will thereby be blinded to the intervention.

Data for statistical analyses and conclusion drawers will also be blinded with the two intervention groups coded as, e.g. X and Y. The steering committee will write two abstracts while the blinding is intact, one assuming the experimental intervention group is X and the control intervention group is Y, and one assuming the opposite. After this, the code should be broken.

### Trial sites

The preGallStep trial has been initiated by the Digestive Disease Centre at Bispebjerg-Frederiksberg Hospital (BFH) and the Surgical Department at Amager-Hvidovre Hospital (AHH). Subsequently, the Department of Surgery at Regional Hospital Aabenraa (RHA) and the Department of Surgery at Regional Hospital Horsens (RHH) will begin the inclusion. More trial sites may be included if they meet the required level of expertise of minimum 20 laparoscopic common bile duct explorations or 200 ERCs [29].

### Trial interventions

#### Experimental intervention—the one-step approach

The one-step approach entails laparoscopic common bile duct exploration (LCBDE) plus laparoscopic cholecystectomy (LC) performed under generalised anaesthesia. Once the dissection has exposed the cystic duct, a clip or ligature is placed peripherally on the cystic duct. Through an incision in the duct proximal to the clip or ligature, a cholangiogram catheter is introduced, and the cholangiogram is completed. After identification of the CBDS and anatomy, a cholangioscope is introduced into the common bile duct through the cystic duct incision. The stones are identified and removed with a Dormia basket. If the stones are very large, they may be fractioned mechanically or by electrohydraulic lithotripsy. In the presence of CBDS wedged in the papilla, these stones will be removed through the mentioned measures or pushed into the duodenum. Subsequently, the cholangioscope is taken out. The cystic duct is divided, and the gallbladder is removed.

#### Control intervention—the two-step approach

The two-step approach entails ERC with sphincterotomy and stone extraction (first step) plus a subsequent laparoscopic cholecystectomy (LC) (second step). ERC is routinely performed in sedation but can also be performed in generalised anaesthesia. ERC is performed with the patient in the supine position. The duodenoscope is passed down to the second part of the duodenum where the papilla is identified. Cannulation of the papilla and the common bile duct is performed with a papillotome and a guide wire. A cholangiogram will confirm the presence, location and size of the CBDS and will aim in the choice of extraction method. Stones can be extracted through the papillotomy by either a balloon or basket. Additional balloon dilation of the papilla, or lithotripsy may be required. Finally, a full cholangiogram is performed through a balloon catheter and a picture is taken for documentation of a clear common bile duct. If stone extraction is incomplete or if the conditions are unclear, a temporary common bile duct stent is placed which must be removed by an additional ERC after 1 to 3 months.

In a separate procedure, the subsequent LC is carried out 2 to 14 days after the initial ERC.

### Outcomes

#### Primary feasibility outcomes

We wish to explore the feasibility of a future large pragmatic randomised clinical trial by evaluating the demand, implementation, practicality and adaptation of the preGallstep trial in a clinical setting. We will assess the following in a qualitative manner:

- Consumption of manpower
- Difficulties getting the first participant randomised at each clinical site
- Reasons for rejection to participate
- Reasons for not being eligible for inclusion
- Difficulties during the informed consent procedure
- Difficulties with randomisation
- Difficulties in data management
- Difficulties with blinding patient charts and forms
- Difficulties in maintaining blinding for the outcome assessors

We will assess the following in a quantitative manner:

- Proportion of eligible patients not wanting to participate.

#### Secondary pilot outcome

- The proportion of participants in both interventional groups with at least one postoperative complication during the 90 days of follow-up, assessed according to the Clavien-Dindo score grade II and above.

#### Exploratory clinical outcomes

This pilot trial will, furthermore, explore the effects of the interventions on several clinical outcomes:

- Common bile duct stone size
- Number of common bile duct stones
- Anatomical position of common bile duct stones
- Length of hospital stay (days)
- Procedure-specific outcomes stone clearance failure, number of procedures needed, method of extraction, procedure time and conversion rate
- Quality of life (SF-36)
- Liver biochemistry

Furthermore, we will explore the distribution of participants according to the highest grade of the Clavien-Dindo score (0; I; II, III; IV; V) in each group.

All exploratory findings will be interpreted conservatively.

#### Sample size

This is a feasibility and pilot trial assessing the possibility of conducting a large-scale, pragmatic randomised clinical trial with the same patient-related outcome. Thus, no formal sample size estimation has been conducted. We pragmatically aim to include 150 participants in total, 75 in each group. With the current numbers of procedures performed at each institution per year, the necessary trial inclusion time to include patients is approximately 18 months.

#### Central data monitoring

Central data monitoring will be initiated after the inclusion of one third of the participants and carried out every third month by a central data monitoring group consisting of investigators, including experienced clinicians, statisticians and trialists. The aim of the central data monitoring is to optimise completeness and quality and minimise deviations through blinded evaluation of the data [30].

Before the initiation of central data monitoring, we will publish a detailed central data monitoring plan including a description of the process.

#### Data analysis

Statistical analysis will be conducted according to the intention-to-treat principle, and analyses will be adjusted for the stratifying variable “site” only. Since all participants included in the trial have CBDS according to the inclusion criteria and according to the best diagnostic modalities used in the clinic today (see above), the intention-to-treat principle can be used without any post-randomisation exclusions. Thereby, all patients with spontaneously passed stones at intra-operative cholangiography, lost to follow-up at 90 days or patients not undergoing the full two-step procedures will be included in the statistical analyses.

Feasibility outcomes will be assessed using descriptive statistics with the use of different types such as measures of variability and distribution.

Our secondary pilot outcome will be blindly assessed and analysed using logistic regression.

All continuous exploratory clinical outcomes will be analysed by linear regression, and other dichotomous exploratory clinical outcomes will also be analysed using logistic regression. Analyses will be performed in Stata (StataCorp LLC, Texas, USA), SAS (SAS Institute, North Carolina, USA) and/or R (R Core Team, Vienna, Austria).

Before the randomisation of the last participant, we will develop and publish a detailed statistical analysis plan with a detailed description of the analyses.

Due to the relatively small sample size, we will not conduct interim analyses. Thus, we will not employ a data monitoring committee.

All results will be presented with 95% confidence intervals (CI).

#### Ethics

The preGallStep trial will be conducted in compliance with the present protocol and the Helsinki Declaration [31]. The preGallStep trial also complies with the requirements from the Regional Ethics Committee of the Capital Region (H-20041609, 4 March 2021) and the Danish data protection laws (“databeskyttelsesforordningen” and “databeskyttelsesloven”) (P-2020-1056, 13 November 2020). The protocol was registered on ClinicalTrials.gov prior to the inclusion of the first participant (NCT04801238, 16 March 2021). Any substantial deviation from the protocol will only be implemented after review and approval from relevant regulatory authorities.

#### Safety

Both treatment strategies in the preGallstep trial are well-established treatments for CBDS and have been tested in the clinic with several observational studies and randomised trials in both Europe and in other continents and both techniques are, therefore, considered safe [32].

#### Adverse events

Adverse events will be noted in the patient medical chart. The surgeon on call will take relevant reactions to the event. At 90 days of follow-up, the coordinating investigator will assess if any serious adverse events (SAE), serious adverse reactions (SARs) or suspected unexpected serious adverse reactions (SUSARs) have occurred in order to report these to the regional ethics committee within 7 days of receiving the information.

## Discussion

The preGallstep trial is the first randomised clinical trial with blinded outcome assessment comparing two different approaches to CBDS. Our focus is not just to compare the two different approaches to CBDS, but also to focus on patient-reported outcomes.

This randomised feasibility and pilot clinical trial have a pragmatically chosen sample size, with associated risks of random errors in the assessment of complication estimates. Yet, we have based our estimates for this feasibility and pilot trial on previous trials and on the flow of relevant patients through the surgical departments in Denmark.

The variations in the inclusion procedures of each trial site due to patients being both acute and ambulant could cause one of the bigger challenges in estimating feasibility issues and implementing changes in procedure in a larger pragmatic trial. We will implement a central data monitoring scheme designed to illustrate “inter-site” differences and the causes of these [30]. These differences are both regarding screening and randomisation, but also executing the trial interventions and follow-up. This monitoring plan will be published separately to ensure complete transparency.

We have not established a priori threshold to judge whether to proceed with a future definitive trial. There are many external factors that influence the conduction of a multicentre RCT such as regional politics and changes in clinical treatment guidelines. Therefore, our focus is not to pragmatically define a threshold but to evaluate these regional differences in planning a future trial. Also, there may be differences in handling complications to surgery at trial sites. Consequently, differences in the Clavien-Dindo score assessments among sites are expected. This may affect the generalisability of this feasibility trial.

The data collected in the present trial will not be pooled with data for a subsequent larger pragmatic trial since there will be differences in procedures. However, the data from the present trial will be available for later systematic reviews with meta-analysis.

The Danish guidelines for the treatment of gallstone disease were published in 2006 [33], and an interdisciplinary group of clinicians has been working on a revision since 2021. In the revised guidelines [34], a one-step procedure is recommended for the treatment of CBDS. This is also recommended in the ESGE guidelines from 2019 [3]. Both guidelines recommend one-step procedures without discriminating between laparoscopic common bile duct exploration or rendez-vous endoscopic retrograde cholangiography during laparoscopic cholecystectomy. The recommendations for rendez-vous is, however, weak with each trial underpowered [34]. As shown in the present study, this also holds for an updated meta-analysis of the question. In the ESGE guidelines, it is emphasised that implementing rendez-vous ERC does carry logistical problems related to the prolonged surgical times and the need to perform ERC in an environment that is not adapted for endoscopy.

In this protocol, we have provided the background and rationale for conducting a trial that compares one-step versus two-step procedures for the removal of CBDS. We have shown an insufficient sample size of previously conducted randomised trials according to TSA. Although there is a tendency to recommend a one-step procedure by some, it is still important to explore the feasibility of implementing such treatments in the clinical setting as a try to obtain better evidence for the recommendations in our clinical guidelines.

## Data Availability

After the results have been published, we aim to make a depersonalised dataset publicly available on, e.g. ClinicalTrials.gov and/or the ZENODO database. The final choice will reflect which platform(s) that are compliant with current legislation at that time.
